# The Effects of Anticipating Metacognitive Judgments about Mind-Wandering

**DOI:** 10.3758/s13414-026-03228-5

**Published:** 2026-02-14

**Authors:** Adrian B. Safati, Daniel Smilek

**Affiliations:** Department of Psychology, University of Waterloo, Waterloo, Ontario Canada

**Keywords:** Mind-wandering, Metacognition, Anticipation, Experience sampling, Attention regulation, Thought probes

## Abstract

Across two experiments (*N* = 121, 121) we examined whether the anticipation of metacognitive judgments in the form of experience sampling probes of mind-wandering influenced attentional engagement. Participants completed a metronome response task in which they tried to hit the spacebar in sync with a steady metronome tone. To maximize possible anticipatory effects, we implemented a task condition in which a visually presented timeline explicitly indicated the precise moments when participants would be required to report on their experiences of mind-wandering. We compared this condition to one in which thought probes were presented without any visual cues signaling when they would appear. Results demonstrate that the visual presentation of when experience sampling events would occur produced significant reductions in mind-wandering and decreases in task performance as the probe approached. These findings suggest that the anticipation of metacognitive judgments reallocates cognitive resources, suppressing both mind-wandering and task-related processing. More broadly, these results demonstrate that the anticipation of metacognitive demands can play an active role in the regulation of attentional processes. The deidentified experimental data, analysis code, and study materials are available at this link https://osf.io/phjkq/?view_only=e521db50368b4295a939c66ce1db2162.

## Introduction

It is rarely the case that people can allocate their full attention to a mundane task for a prolonged period of time (Cheyne et al., 2006; Mackworth, 1948; Robertson et al., 1997; Smilek et al., 2010b). Indeed, the evidence suggests that people are often distracted by task-unrelated internal thoughts (Smallwood et al., 2003) or tasks other than the one they have committed to perform (Monsell, 2003; Ralph et al., 2014). Over time, attention to a task fluctuates (Pereira et al., 2024) and often declines (Mackworth, 1948; Risko et al., 2012; Thomson et al., 2014; Zanesco et al., 2024). While the dynamics of attention can be, to some extent, inferred from task performance (e.g., Seli, Cheyne, et al., 2013a, 2013b) and physiological measures (e.g., Fox et al., 2015; Kam et al., 2022), these only index attention indirectly. Since attentional shifts are marked by (and even defined by) changes in consciousness, a more direct way to index attentional engagement and disengagement is by using various experience sampling techniques that directly ask participants to report on their attentional focus (e.g., Giambra, 1995; Schooler et al., 2011). Within research assessing attentional engagement (e.g., inattention, distraction), this has involved using intermittent “thought probes” that interrupt an ongoing task and require a participant to make metacognitive judgments about the nature of their attentional state before the probe appeared (Jackson & Balota, 2012; Risko et al., 2012; Schubert et al., 2020; Stawarczyk et al., 2011; Weinstein, 2018). These thought probes are repeated during the progression of the primary task, allowing for multiple samples of attentional states throughout the course of the task.

A good example of this experience sampling technique comes from studies of “mind-wandering” (Seli, Kane, et al., 2018a, 2018b; Smallwood & Schooler, 2015), or in other words, task-unrelated thought (Giambra, 1995; Seli et al., 2015a, 2015b; Smallwood et al., 2003). For instance, Giambra (1995) had participants complete simple attention tasks on a computer in which individuals were presented with either more frequent “neutral” events (e.g., a green circle) or less frequent “target” events (e.g., three red squares). Participants were instructed to quickly respond whenever one of the target stimuli appeared. As the task progressed, participants were interrupted with tones indicating they should report whether they experienced any task-unrelated imagery or thoughts in the prior 29 s of the task (i.e., in the trials following the previous tone).

Since these early studies, researchers have used many variants of the experience sampling procedure to assess attentional engagement and disengagement within a given task context (see Weinstein, 2018). Thought probes have varied in terms of: (1) whether they ask people the extent to which their thoughts were off-task (e.g., Smilek et al., 2010a) or on-task (e.g., Jackson & Balota, 2012); (2) whether the probes ask about general levels of inattention (e.g., Risko et al., 2012), or more specific aspects, such as whether a mind-wandering episode occurred deliberately (intentionally) or spontaneously (unintentionally) (e.g., Seli et al., 2016); (3) whether they allow for categorical (e.g., Seli et al., 2015a, 2015b) or continuous (e.g., Macdonald et al., 2011) responses; (4) whether they occur at fixed intervals (e.g., Giambra, 1995) or at varying intervals (e.g., Seli, Carriere, et al., 2013a, 2013b); and (5) whether they ask about attention over a longer interval (e.g., Farley et al., 2013; Giambra, 1995) or a shorter interval (e.g., *just before* the probe appeared; Christoff et al., 2009). Regardless of these variations, the general assumption has been that responses to these thought probes accurately reflect people’s attentional allocation in the given context. Consistent with this assumption are many studies that show a convergence between people’s subjective responses to thought probes and objective task performance, whereby in most cases, increased reports of task inattention have been linked to decreased task performance (Anderson et al., 2021; Feng et al., 2013; Mooneyham & Schooler, 2013; Randall et al., 2014; Safati et al., 2024; Seli, Cheyne, et al., 2013a, 2013b; Wammes et al., 2016; Yanko & Spalek, 2014).

One fundamental issue that has emerged from this growing body of work is whether the thought sampling probes serve not only as indices of (in)attention but also influence levels of (in)attention to a primary task. While the presence or absence of probes does not necessarily impact task performance (Wiemers & Redick, 2019), it has been shown that reports of mind-wandering are influenced by the frequency of (i.e., duration between) thought probes within a task (Safati et al., 2024; Schubert et al., 2020; Seli, Carriere, et al., 2013a, 2013b). The general finding is that as the interval between thought probes decreases, reports of mind-wandering also decrease. A possible explanation of this pattern is that thought probes reorient attention to the primary task and when thought probes are presented more closely together in time, there is less time for the mind to wander before the next probe appears. While increasing probe frequency does not seem to meaningfully undermine the commonly found relations between probe reports and performance (Schubert et al., 2020), the findings do suggest that the thought probe method can influence reports of attention within a given task context.

Further along these lines, given that thought probes are often repeating events within the experimental context, when seeking to understand the potential impacts of thought probes we should consider how *anticipation* of task events influences attention and performance. While there are several domains in the cognitive literature in which anticipation has been investigated (Clark, 2013; Karlin, 1959; Kunde et al., 2012; Los et al., 2017; Niemi & Näätänen, 1981), here we focus on one recent example from the mind-wandering literature. Seli et al. (2018a, 2018b) had participants track a clock hand moving around a clock and respond with a button press every time the hand struck 12 o’clock. The hand moved at a consistent speed and participants responded to thought probes at different times as the hand moved around the clock. The results showed that mind-wandering rates were higher when the clock hand was further away from 12 o’clock and lower as it approached and followed 12 o’clock. These results imply there was a strategic refocusing of attention to the task and away from mind-wandering as a response became imminent. Given these findings, it is reasonable to speculate that anticipation of an introspective event – the upcoming response to a thought probe – might also change the focus of attention within the task context. In other words, the presence of thought probes could actively shape attentional engagement rather than merely measure it.

How might anticipation of an upcoming thought probe influence attention and performance? One possibility is that anticipating an upcoming probe might increase motivation to focus attention on the primary task, thus improving task attention and performance. Indeed, several studies have shown that higher task motivation is associated with reduced mind-wandering and improved performance (Brosowsky et al., 2020; Seli et al., 2019; Unsworth & McMillan, 2013). Furthermore, in cognitive tasks with clearly defined boundaries, researchers examining goal pursuit behaviors have often observed increased motivation and performance near starting and ending states (Bonezzi et al., 2011). This phenomenon has been observed across a wide range of field and lab experiments and is thought to result from separable motivations to (i) reach the end state, and (ii) maintain a positive self-image of having done the task well upon completion (Touré-Tillery & Fishbach, 2012). Like end states, intermittent thought probes may function as motivational checkpoints, where the anticipation of an upcoming probe encourages participants to refocus their attention on the task.

A possible alternative effect of anticipating a thought probe emerges from a consideration of the cognitive processing involved in responding to a thought probe. Thought probes asking individuals to introspect about their attentional processes can be construed as involving a “metacognitive judgment” (i.e., thinking about one’s cognitions; Dunlosky & Metcalfe, 2008; Flavell, 1979; Norman et al., 2019) that require individuals to evaluate their “metacognitive experience” (Flavell, 1979). Because these judgments evaluate one’s attentional focus, we can refer to these as “meta-attentional” judgments (see Loper & Hallahan, 1982). Such judgments entail being aware of the outcome of a process that monitors consciousness, with such awareness itself depending on limited resources (Schooler, 2002). It follows then, that anticipating a thought probe might divert limited attentional resources from both mind-wandering and task performance, applying them instead to meta-attentional evaluation. This would lead to a seemingly paradoxical effect whereby anticipating an imminent thought probe could reduce mind-wandering and decrease performance in the moments leading up to the presentation of the thought probe.

Herein, we aimed to investigate whether and how anticipating the presentation of a metacognitive thought probe about attentional engagement influences probe responses and task performance. To maximize possible anticipatory effects, we implemented a condition in which a visually presented timeline of the experimental task explicitly indicated the precise moments when thought probes would occur during a primary attention ask. We compared this condition to one in which thought probes were presented without any visual cue signaling their onset. By systematically manipulating whether probe onset is perfectly predictable or not, we were able to examine whether and how anticipation influences thought probe responses and performance in the attention task. If anticipation does affect reported mind-wandering rates and/or performance, this would provide valuable insights into basic mechanisms underlying meta-attentive judgments and the dynamic regulation of attentional engagement.

## Experiment 1

**Fig. 1:**
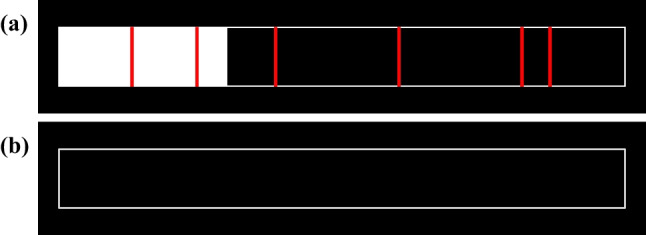
The between-group manipulation in Experiment 1. Note: Panel (**a**) shows the top of the black screen in the Visible condition. A centered rectangle indicated the timeline of the experimental task; red lines marked the time points when thought probes would appear; and a white progress bar advancing from the left to the right indicated how much relative time had elapsed. Panel (**b**) shows the top of the screen in the Not Visible condition, which included an outline of a static rectangle at the top of the black screen that did not provide any information about the temporal pattern of the thought probes or task progress

The purpose of Experiment 1 was to examine whether anticipation of an upcoming thought probe about one’s mind-wandering influences probe responses and performance. Participants in the study completed the Metronome Response Task (MRT; Anderson et al., 2021; Safati et al., 2024; Seli, Cheyne, et al., 2013a, 2013b), which involves pressing a button in tempo with a repeated tone presented at equal intervals. Throughout the MRT participants responded to thought probes about their levels of mind-wandering. The thought probes included ratings of both deliberate and spontaneous mind-wandering to more comprehensively measure different aspects of mind-wandering (Robison & Unsworth, 2018; Seli, Carriere, et al., 2015a, 2015b; Seli et al., 2016). Critically, we employed a between-subjects design, assigning participants to either the “Visible” or the “Not Visible” probe timing group. Figure 1a illustrates how participants in the “Visible” group were shown a progress bar at the top of the screen with clear red lines along the route of the progress bar indicating the precise moments when thought probes would appear. Figure 1b illustrates how participants in the “Not Visible” group were not shown when the thought probes would appear and were only shown a static rectangle at the top of the screen (i.e., without the progress bar).

If anticipating responding to a thought probe influences attention and performance, there could be several possible outcomes. First, we might expect that participants in the Visible probe condition might report lower rates of mind-wandering and exhibit improved task performance relative to those in the Not Visible condition, especially leading up to the moment a probe is presented. Such a result would suggest that foreknowledge of probe onset serves as a motivational cue, encouraging participants to maintain focus on the primary task as the probe approaches. Alternatively, probe anticipation might increase meta-attentional monitoring, thus reducing mind-wandering and performance in the moments before the probe in the Visible probe condition relative to the Not Visible probe condition. Finally, if probe anticipation does not meaningfully affect attentional allocation, we would expect no significant differences in reported mind-wandering or task performance between conditions.

### Methods

Our research was approved by the Office of Research Ethics at the University of Waterloo. The deidentified experimental data, analysis code, and study materials are available at this link: https://osf.io/phjkq/?view_only=e521db50368b4295a939c66ce1db2162.

#### Participants

A target sample size of 120 was chosen to ensure detection of medium-sized between-group effects. A total of 123 participants were recruited from the University of Waterloo undergraduate SONA research pool to participate in a 30-min study. Two participants were excluded from the analysis due to a high number of missed responses (> 15%). The analyzed sample (*N* = 121) consisted of 99 women (cis or trans), 21 men (cis or trans), and one non-binary participant, and with ages ranging from 18 to 36 (*M* = 19.97, *SD* = 2.55) years. Participants were randomly assigned to either the “Visible” group (*N* = 62) or the “Not Visible” group (*N* = 59).

#### Materials

Metronome Response Task (MRT). In the MRT, participants heard repetitive auditory tones resembling a metronome beat. They were asked to synchronize their spacebar presses with the beginning of each tone, requiring them to anticipate rather than simply react to the sound. The duration of each trial totaled 1,300 ms, beginning with 650 ms of quiet, followed by a 75-ms tone, and ending with an additional 575 ms of silence. Periodically, the MRT was interrupted by a thought probe presented at unpredictable intervals, prompting participants to indicate their current level of mind-wandering. After completing a probe response, participants continued with the task. Performance on the task was assessed using a measure called “rhythmic response time variability” (Seli, Cheyne, et al., 2013a, 2013b). This measure was computed based on deriving the temporal difference between a spacebar press and its corresponding metronome tone onset for each trial. Trials with absent responses were excluded, and the rhythmic response time variability (RRTv) was determined by calculating variance across consecutive five-trial runs. To address the positive skew of the data and achieve better normality, a natural-log transformation was implemented. This analytical approach mirrors the methods used in previous MRT research (Seli, Cheyne, et al., 2013a, 2013b).

During the task the computer screen was black and contained a white outline of a rectangle that was centered at the top of the screen (see Fig. 1). In one condition (the Visible condition) the horizontal aspect of the white rectangle outline was meant to depict a timeline of the MRT task. In this condition, a white progress bar advanced from the left to the right side of the outlined rectangle to indicate how much relative time had elapsed in the task (and how much relative time was left), and red vertical lines marked the time points when the thought probes would be presented (see Fig. 1a). Participants were told that whenever the progress bar touched a red marker a probe would appear. In the other condition (the Not Visible condition), only the horizontal white rectangle outline was shown; there was no progress bar and no red lines marking when probes would be presented (see Fig. 1b). Participants were told that they would see a rectangle at the top of the screen, but the relevance of the rectangle to the experiment was withheld until they had completed the task.

*Mind-wandering thought probes.* As previously described, the MRT was interrupted by thought probes at random intervals; individuals needed to complete the thought probes before proceeding with the task. Each thought probe consisted of two visual analogue scales displayed on a single screen. Participants were prompted to respond to “How much were you mind-wandering...” (i) “deliberately right before this probe appeared?” and (ii) “spontaneously right before this probe appeared?” Mind-wandering was defined for participants as episodes where their thoughts drifted away from their current task or immediate environment to a loose internal stream of consciousness. Participants were given the examples of mind-wandering that might occur in daily life such as while reading a book, going for a walk, or sitting in a lecture having their thoughts drift away from what they were doing and what was going on around them to topics like the TV show they watched last night, their plans for dinner, or what it means to be happy. Participants were told that mind-wandering could be spontaneous/unintentional, as in the case of trying to attend to a lecture but finding your thoughts drifting off despite your intentions; or that mind-wandering could be deliberate/intentional, as in the case of trying to block out and disconnect from a lecture that was repeating information that they already knew. Participants were told that while there could be some overlap between spontaneous and deliberate mind-wandering, they should try their best to distinguish between the two and report on their mental state as accurately as possible. The presentation order of the deliberate and spontaneous scales was randomized across participants and the end of each scale was anchored with “*Not at all*” on the left and “*A lot*” on the right.

#### Procedure

The study was conducted in-person in laboratories at the University of Waterloo. Multiple participants were tested concurrently in the same room with dividers separating the computers. Participants were randomly assigned to either the Visible or the Not Visible condition. Upon entering the testing room, the participants were seated in front of an assigned computer and provided with instructions for the experimental session. After completing the information consent letter, and a brief demographic questionnaire to characterize the sample, participants completed the metronome response task.

The present implementation of the MRT consisted of a practice block of 60 trials followed by three experimental blocks of 300 trials each (900 trials total). Each experimental block lasted approximately 8 min, varying slightly by how quickly participants responded to probes. The number of trial responses and average response times during the practice block were assessed during data collection to ensure participant understanding of task expectations before continuing the task. Each of the three experimental blocks had six probes distributed throughout, with probes assigned to appear pseudo-randomly between trials 10–50, 60–100, 110–150, 160–200, 210–250, and 260–300. The pseudo-random distribution of probes was done to ensure a distributed spread of the probes throughout the block with sufficient spacing between individual probes. Throughout the experiment each participant completed a total of 18 probe reports in addition to the two in the practice block. Participants had the opportunity to practice answering two probes during the practice block of the task appearing at trials 20 and 40 of 60. After all participants in a scheduled session finished the task, they were debriefed and thanked for their participation.

### Analysis

We employed linear mixed-effects models to statistically assess group condition effects, utilizing the lme4 package (Bates et al., 2014) in R version 4.3.3 (R Core Team, 2024). Table 1 presents comprehensive model summaries that include model specifications, observation counts, fit statistics, and ANOVA findings for each predictor. Model 1.1 analyzed reports of both spontaneous and deliberate mind-wandering as the outcome variable, whereas Model 1.2 focused on MRT RRTv as the dependent measure. We verified model assumptions using the performance package (Lüdecke et al., 2021). For post hoc comparisons, we applied the Kenward-Roger approximation for degrees of freedom through the emmeans package (Lenth, 2023). The ANOVA results shown in Table 1 were evaluated for significance using the Satterthwaite degrees of freedom approximation implemented in the lmerTest package (Kuznetsova et al., 2017).

**Table 1:** ANOVA tables for thought probe reports and task performance

Model	Parameter	*SS*	*MS*	*df*_*Num*_	*df*_*Den*_	*F*	*p*	*η*^2^
Model 1.1	Model Formula: Mind_Wandering ~ condition*subtype + (1 | participant:subtype) Model Data: Observations = 4356, Performance: *R*^*2*^_*Marginal*_ = 0.070, *R*^*2*^_*Conditional*_ = 0.569							
	condition	2308.25	2308.25	1	238	5.918	0.016	0.02
	subtype	9383.30	9383.30	1	238	24.056	<0.001	0.09
	condition:subtype	1095.87	1095.87	1	238	2.809	0.095	0.01
Model 1.2	Model Formula: LN_RRTv ~ condition*distance + (1 | participant) Model Data: Observations = 20474, Performance: *R*^*2*^_*Marginal*_ = 0.004, *R*^*2*^_*Conditional*_ = 0.312							
	condition	2.15	2.15	1	123.2	1.761	0.187	0.01
	distance	71.55	71.55	1	20351.2	58.665	<0.001	<0.01
	condition:distance	42.10	42.10	1	20351.2	34.519	<0.001	<0.01

Model 1.1 (Table 1) examined how visibility of when the probes would appear {“Visible”, “Not Visible”} influenced reports of mind-wandering, considering differences in the type of mind-wandering report {“spontaneous” or “deliberate”} and the interaction between these terms. In the fixed effects we examined the interaction between task condition and mind-wandering subtype. Our random effects specified random intercepts for spontaneous and deliberate mind-wandering reports as these are distinct constructs.

Model 1.2 (Table 1) examined how probe marker visibility {“Visible”, “Not Visible”} influenced response time variability, and whether an interaction emerged in the trials closest to the probe. We categorized distance using a binary predictor {“Proximal” or “Distal”}, where response variability scores were defined as “Proximal” if they were in the window immediately preceding the probe and “Distal” if they occurred before the “Proximal” window. Response variability scores are calculated using discrete contiguous windows of five trials, which encompass a period of 6.5 s (i.e., 5 × 1.3 s per trial). In the fixed effects we examined the interaction between task condition and distance from the probe. Our random effects specified random intercepts for each participant.

### Results

#### The effect of probe marker visibility on mind-wandering reports

**Fig. 2:**
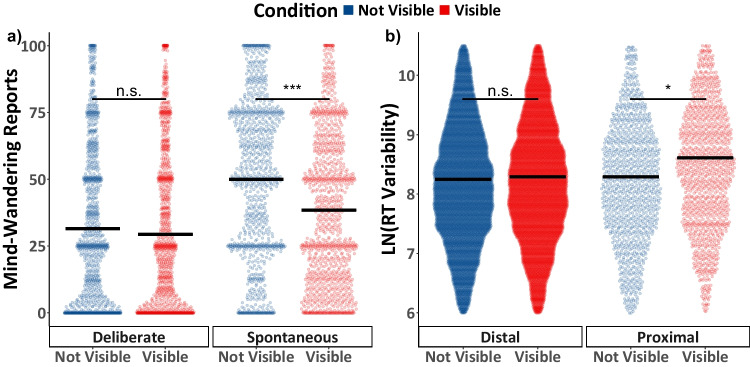
Experiment 1 mind-wandering and task performance results. In panel (**a**) the colored points illustrate all raw participant responses to the mind-wandering probes, in panel (**b**) the colored points illustrate response time (RT) variance scores within the selected range. In panel (**b**) RT variability scores from the five-trial window immediately preceding the probe are labelled as “Proximal”; other trial windows were classified as “Distal.” The estimated marginal means from Model 1.1 for panel (**a**), and from Model 1.2 for panel (**b**) are presented in black. Pairwise comparisons with significance scores are shown above the marginal means, * = *p* < 0.05, ** = *p* < 0.01, *** = *p* < 0.001

To examine how being shown when the probes would appear influences mind-wandering, both spontaneous and deliberate mind-wandering reports were fit using Model 1.1 (see Table 1). The data along with comparisons of the estimated marginal means are shown in Fig. 2a. The interaction between task condition and mind-wandering subtype in Model 1.1 was not significant, *F*(1, 238) = 2.809, *p* = 0.095, *η*2 =.01. However, there was a significant main effect of task condition, *F*(1, 238) = 5.918, *p* = 0.016, *η*2 =.02, such that mind-wandering reports were greater in the Not Visible condition relative to the Visible condition, *t*(238) = 2.43, *p* = 0.016, *d* = 0.35. There was also a significant main effect of mind-wandering subtype, *F*(1, 238) = 5.918, *p* = 0.016, *η*2 =.09, as spontaneous mind-wandering reports were greater than reports of deliberate mind-wandering, *t*(238) = 4.91, *p* < 0.001, *d* = 0.70. Exploratory simple-effects analysis of the main effect of task condition revealed that probe marker visibility resulted in a significant reduction in spontaneous mind-wandering, *t*(238) = 2.905, *p* = 0.004, *d* = 0.58, but the effects were not significant for deliberate mind-wandering, *t*(238) = 0.535, *p* = 0.593, *d* = 0.11.

#### The effect of probe marker visibility on task performance

To examine how the visibility of the probes would influence task performance, we fit the response time variability data in Model 1.2 (see Table 1). The model included a binary predictor to distinguish response time variability scores that were “Proximal” to the probe (i.e., from the five-trial window preceding the probe) from those that that were further away (i.e., “Distal”). The data along with pairwise comparisons of the estimated marginal means are shown in Fig. 2b. The main effect of condition on response variability scores was not significant, *F*(1, 123.2) = 1.761, *p* = 0.187, *η*2 =.01, but there was a significant main effect of probe distance, *F*(1, 20351.2) = 58.665, *p* < 0.001, *η*2 <.01, which was qualified by a significant interaction between condition and distance from the probe, *F*(1, 20351.2) = 34.519, *p* < 0.001, *η*2 <.01. Examining the simple effects reveals significant differences in task performance across conditions for trials proximal to the probe, *t*(142) = 2.261, *p* = 0.025, *d* = 0.29, but not for trials distal to the probe *t*(119) = 0.312, *p* = 0.755, *d* = 0.04.

### Discussion

The precise knowledge of when experience sampling probes would appear significantly reduced reports of mind-wandering, with this effect occurring primarily for spontaneous but not deliberate mind-wandering. Examining task performance, we observed a significant decrease in performance in the window of five trials immediately preceding indicated probes. Typically, reductions in mind-wandering are associated with improved task performance (Anderson et al., 2021; Mooneyham & Schooler, 2013; Randall et al., 2014; Safati et al., 2024; Seli, Cheyne, et al., 2013a, 2013b), but here we observed lower mind-wandering coupled with reduced performance. The pattern of results suggests that the anticipation of making meta-attentional judgments pulled limited mental resources from both mind-wandering and the cognitive task.

## Experiment 2

**Fig. 3:**
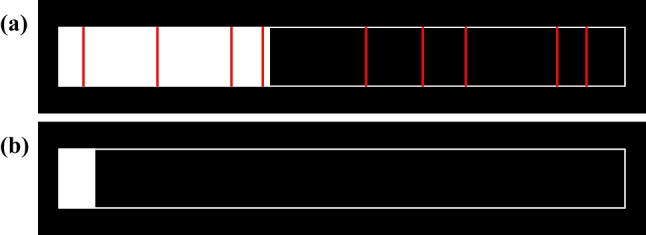
The between-group manipulation in Experiment 2. Panel (**a**) shows the top of the black screen in the Visible condition, which included a white rectangle indicating the timeline, red lines marking the time points at which a probe would appear, and a solid white rectangle progressing from left to right indicating the amount of time elapsed. Panel (**b**) shows the top of the screen in the Not Visible condition. The solid white rectangle progressed from left to right to indicate the amount of time elapsed. There were no red lines indicating when the probes would appear

The aim of Experiment 2 was twofold. First, we sought to investigate the robustness of the outcomes of Experiment 1. To this end, Experiment 2 again included two conditions, with one condition, illustrated in Fig. 3a, containing visual cues signaling upcoming thought probes (the “Visible” probe condition) and the other condition, illustrated in Fig. 3b, excluding visual cues (the “Not Visible” probe condition). This time, however, participants experienced both conditions in a counterbalanced order, using a within-participant design to gain more power to detect effects between conditions. Second, we sought to gain a more nuanced understanding of potential anticipation-related shifts in internal thought. Accordingly, we included in our thought probe other dimensions of thought, such as thoughts about future aspects of the task, or concerns about task performance. Anticipation-related changes in responses to these dimensions would suggest that probe anticipation could alter the content of task-relevant conscious thought. Alternatively, if no effects of anticipating probes emerged in these additional dimensions of thought, yet overall inattention and performance decreased as a function of anticipation, this would indicate that any effects of probe anticipation are specific to the general process of metacognitive monitoring rather than the nature of specific thoughts during the process.

### Methods

#### Participants

A target sample size of 120 was selected to allow for the detection of small within-group effects. A total of 122 participants were recruited from the University of Waterloo undergraduate SONA research pool to participate in a 30-min study. One participant was dropped from the analysis due to a high number of missed responses (> 15%). The analyzed sample (*N* = 121) for Experiment 2 consisted of 89 women (cis or trans), 27 men (cis or trans), and five non-binary participants with ages ranging from 17 to 37 (*M* = 19.77, *SD* = 2.46) years. In a within-subject design participants were randomly assigned to either complete the “Visible” condition first (*N* = 59) or the “Not Visible” condition first (*N* = 62). Participants completed one experimental block corresponding to each condition.

#### Materials

MRT. The design of the MRT was slightly modified from Experiment 1. In place of the three experimental blocks of 300 trials used in Experiment 1, in Experiment 2 there were two experimental blocks of 450 trials (900 trials total), this modification allowed for simpler balancing of the two task conditions. Due to the change in trial distribution, the longer experimental blocks in Experiment 2 lasted approximately 13 min.

During the task the computer screen was black and contained a white outline of a rectangle that was centered at the top of the screen (see Fig. 2). In both conditions the horizontal aspect of the white rectangle outline was meant to depict a timeline of the MRT task, as a white progress bar advanced from the left to the right side of the outlined rectangle to indicate how much relative time had elapsed in the task (and how much relative time was left). In the Visible condition red vertical lines marked the time points when the thought probes would be presented (see Fig. 2a). Participants were told that whenever the progress bar touched a red marker a probe would appear. In the other condition (the Not Visible condition), the red markers were not displayed but the advancing white progress bar still indicated the relative passage of time for the block (see Fig. 2b).

*Mind-wandering thought probes.* In Experiment 2 each thought probe consisted of four visual analogue scales displayed on a single screen. Using the scales participants were prompted to respond to “Right before this screen appeared to what extent were you…” (i) “mind-wandering deliberately,” (ii) “mind-wandering spontaneously,” (iii) “thinking about future aspects of the task,” (iv) “thinking about how well you are doing in the task?” The concept of mind-wandering and the spontaneous versus deliberate subtypes of mind-wandering were explained using the same definitions and accompanying examples for participants as in Experiment 1. The presentation order of the scales was randomized across participants and the end of each scale was anchored with “*Not at all*” on the left and “*A lot*” on the right.

#### Procedure

Data collection for Experiment 2 followed the same in-person data collection procedures as Experiment 1 with a few notable exceptions to the metronome response task. During the initial practice block and the “Visible” block participants were shown a progress bar at the top of the screen with clear red lines along the route of the progress bar indicating the precise moments when thought probes would appear. In the “Not Visible” block participants were shown the progress bar without the probe markers. The key difference from Experiment 1 was that with the within-subject design of Experiment 2, participants completed one experimental block of each task condition instead of completing multiple blocks of the same task condition. Participants were randomized to the condition they completed first instead of the group to which they were assigned. Each of the two experimental blocks had nine probes distributed throughout, with probes assigned to appear pseudo-randomly between trials 10–50, 60–100, 110–150, 160–200, 210–250, 260–300, 310–350, 360–400, and 410–450. As in Experiment 1, participants completed a total of 18 probes, in addition to the two in the practice block.

### Analysis

Mixed-effects models were used to statistically examine the effect of task condition. Model summaries, including formulas, the number of observations, performance indicators, and ANOVA results for the predictors, can be found in Table 2. The dependent measure of Model 2.1 was reports of spontaneous and deliberate mind-wandering; Model 2.1a examined reports of thoughts about future aspects of the task, Model 2.1b examined reports of thoughts about task performance, while Model 2.2 examined MRT RRTv. The same R analysis packages used in Experiment 1 were used in Experiment 2.

**Table 2:** ANOVA tables for thought probe reports and task performance

Model	Parameter	*SS*	*MS*	*df*_*Num*_	*df*_*Den*_	*F*	*p*	*η*^2^
Model 2.1	Model Formula: Mind_Wandering ~ condition*subtype + order + (1 | participant:subtype) Model Data: Observations = 4356, Performance: *R*^*2*^_*Marginal*_ = 0.062, *R*^*2*^_*Conditional*_ = 0.550							
	condition	17109.65	17109.65	1	4112	41.197	<0.001	0.01
	subtype	10587.05	10587.05	1	239	25.492	<0.001	0.10
	order	741.22	741.22	1	239	1.785	0.183	0.01
	condition:type	572.36	572.36	1	4112	1.378	0.240	<0.01
Model 2.1a	Model Formula: Future_Aspects ~ condition + order + (1 | participant) Model Data: Observations = 2178, Performance: *R*^*2*^_*Marginal*_ = 0.004, *R*^*2*^_*Conditional*_ = 0.506							
	condition	862.45	862.45	1	2056	1.971	0.161	<0.01
	order	339.35	339.35	1	119	0.775	0.380	0.01
Model 2.1b	Model Formula: Task_Performance ~ condition + order + (1 | participant) Model Data: Observations = 2178, Performance: *R*^*2*^_*Marginal*_ = 0.000, *R*^*2*^_*Conditional*_ = 0.603							
	condition	24.07	24.07	1	2056	0.074	0.786	<0.01
	order	17.09	17.09	1	119	0.052	0.820	<0.01
Model 2.2	Model Formula: LN_RRTv ~ condition*distance + order + (1 | participant) Model Data: Observations = 20409, Performance: *R*^*2*^_*Marginal*_ = 0.002,* R*^*2*^_*Conditional*_ = 0.283							
	condition	9.00	9.00	1	20285.0	7.301	0.007	<0.01
	distance	54.39	54.39	1	20285.0	44.138	<0.001	<0.01
	order	0.06	0.06	1	118.9	0.051	0.822	<0.01
	condition:distance	13.95	13.95	1	20285.0	11.316	0.001	<0.01

Models 2.1 (Table 2) examined how visibility of when the probes would appear {“Visible”, “Not Visible”} influenced reports of mind-wandering, considering differences in the type of mind-wandering report {“spontaneous” or “deliberate”} and the interaction between these terms. In the fixed effects we examined the interaction between task condition and mind-wandering subtype, and controlled for the task condition order. Our random effects specified random intercepts for spontaneous and deliberate mind-wandering reports as these are distinct constructs.

Model 2.1a (Table 2) examined how visibility of when the probes would appear {“Visible”, “Not Visible”} influenced reports of thoughts about future aspects of the task. In the fixed effects we examined the effects of task condition and controlled for the task condition order. Our random effects specified random intercepts for each participant.

Model 2.1b (Table 2) examined how visibility of when the probes would appear {“Visible”, “Not Visible”} influenced reports of thoughts about task performance. In the fixed effects we examined the effects of task condition and controlled for the task condition order. Our random effects specified random intercepts for each participant.

Model 2.2 (Table 2) examined how probe marker visibility {“Visible”, “Not Visible”} influenced response time variability, and whether an interaction emerged in the trials closest to the probe. We categorized distance using a binary predictor {“Proximal” or “Distal”}, where response variability scores were defined as “Proximal” if they were in the window immediately preceding the probe and “Distal” if they occurred before the “Proximal” window. Response variability scores are calculated using discrete contiguous windows of five trials, which encompass a period of 6.5 s (i.e., 5 × 1.3 s per trial). In the fixed effects we examined the interaction between task condition and distance from the probe, and controlled for the task condition order. Our random effects specified random intercepts for each participant.

### Results

#### The effect of probe marker visibility on mind-wandering reports

**Fig. 4:**
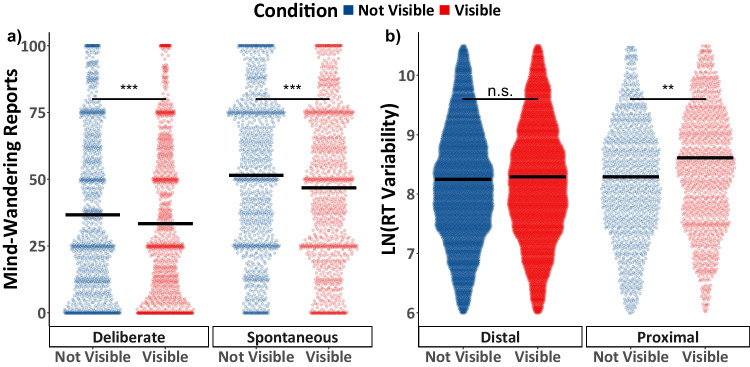
Experiment 2 mind-wandering and task performance results. In panel (**a**) the colored points illustrate all raw participant responses to the mind-wandering probes, in panel (**b**) the colored points illustrate response time (RT) variance scores within the selected range. In panel (**b**), RT variability scores from the five-trial window immediately preceding the probe are labelled as “Proximal”; other trial windows were classified as “Distal.” The estimated marginal means from Model 2.1 for panel (**a**), and from Model 2.2 for panel (**b**) are presented in black. Pairwise comparisons with significance scores are shown above the marginal means, * = *p* < 0.05, ** = *p* < 0.01, *** = *p* < 0.001

To examine how being shown when the probes would appear influences mind-wandering, both spontaneous and deliberate mind-wandering reports were fit using Model 2.1 (see Table 2). Figure 4a shows the data together with comparisons of the estimated marginal means. Analyses revealed that the interaction between task condition and mind-wandering subtype in Model 2.1 was not significant, *F*(1, 4112) = 1.378, *p* = 0.240, *η*2 <.01. There was, however, a significant main effect of task condition, *F*(1, 4112) = 41.197, *p* < 0.001, *η*2 =.01, such that mind-wandering reports were greater in the Not Visible condition relative to the Visible condition, *t*(4112) = 6.419, *p* < 0.001, *d* = 0.20. There was also a significant main effect of mind-wandering subtype, *F*(1, 239) = 25.492, *p* < 0.001, *η*2 =.10, as spontaneous mind-wandering reports were greater than reports of deliberate mind-wandering, *t*(239) = 5.05, *p* < 0.001, *d* = 0.69. The order in which participants completed the task was not found to be a significant predictor in the model, *F*(1, 239) = 1.785, *p* = 0.183, *η*2 <.01. Exploratory simple-effects analysis of the main effect of task condition reveals that the presence of probe timing cues resulted in significant reductions in both spontaneous, *t*(4112) = 5.369, *p* < 0.001, *d* = 0.23, and deliberate, *t*(4112) = 3.708, *p* < 0.001, *d* = 0.16, mind-wandering.

#### The effect of probe marker visibility on other probe dimensions

Relative to Experiment 1, in Experiment 2 we added dimensions to the thought probes. In Model 2.1a (see Table 2) we examined whether the task condition (i.e., whether the probes were Visible or Not Visible) influenced thoughts about future aspects of the task. Participants in the Visible condition (*M* = 34.3, *SE* = 2.02) did not significantly differ from those in the Not Visible condition (*M* = 32.8, *SE* = 2.02), *F*(2056) = 1.971, *p* = 0.161, *η*2 <.01. Likewise, in Model 2.1b (see Table 2) we examined whether the task condition influenced thoughts about task performance. Again, we found no significant differences between the Visible condition (*M* = 35.9, *SE* = 2.12), and the Not Visible condition (*M* = 35.2, *SE* = 2.12), *t*(2056) = 0.271, *p* = 0.786, *η*2 <.01. The order in which participants completed the task was not found to be a significant predictor in either Model 2.1a, *F*(1, 119) = 0.775, *p* = 0.380, *η*2 =.01, or in Model 2.1b, *F*(1, 119) = 0.052, *p* = 0.820, *η*2 <.01.

#### The effect of probe marker visibility on task performance

Finally, we examined how the visibility of a cue indicating when a thought probe would appear influences task performance by fitting the response time variability data in Model 2.2 (see Table 2). As in Experiment 1, the model included a binary predictor of trials that were “Proximal” and “Distal” from the probe. Figure 4b depicts individual data points together with the estimated marginal means for each condition; pairwise comparisons are also illustrated. There was a significant main effect of task condition, *F*(1, 20285.0) = 7.301, *p* = 0.007, *η*2 <.01, and distance from the probe, *F*(1, 20285.0) = 44.138, *p* < 0.001, *η*2 <.01, qualified by a significant interaction between condition and probe distance, *F*(1, 20285.0) = 11.316, *p* = 0.001, *η*2 <.01. The order in which participants completed the task was not found to be a significant predictor in the model, *F*(1, 118.9) = 0.051, *p* = 0.822, *η*2 <.01. Examining the simple effects reveals significant differences in task performance across conditions for the window preceding the probes, *t*(20285) = 3.227, *p* = 0.001, *d* = 0.13, but not for variability scores further from probes, *t*(20285) = 0.969, *p* = 0.333, *d* = 0.01.

### Discussion

Experiment 2 reaffirmed the primary findings from Experiment 1. The presence of visual cues providing information about the precise moments when the thought probes would appear was again associated with significantly reduced reports of mind-wandering, and significantly reduced performance in the window of trials preceding the probe. Unlike Experiment 1, the visible probes were associated with reductions in both spontaneous and deliberate mind-wandering reports. Due to the small effect size observed for the reduction in deliberate mind-wandering, the change in significance between experiments can likely be attributed to the greater power to detect effects with the within-subject design employed in Experiment 2. Regarding other potential domains of thought, we did not observe any significant difference across probe anticipation conditions in reports of *thoughts about future aspects of the task* or *thoughts about task performance*. Given the lack of these differences, it appears that participants are not explicitly thinking about preparing to answer the probes when a visual cue is present signaling the exact time of probe presentation. However, the pattern of results again suggests that the anticipation of making meta-attentional judgments is reallocating mental resources from both mind-wandering and the cognitive task.

## General discussion

The present studies examined whether the anticipation of providing introspective reports about mind-wandering influence individuals’ levels of mind-wandering and task performance. Our experiments involved having participants respond to thought probes about their mind-wandering while performing an attention task under two different conditions. In one condition the precise timing of probe presentation was visually indicated to participants allowing us to maximize the potential for anticipatory changes in response to expected metacognitive demands from the probes. In the other condition participants were not given a visual indication of when the probes would be presented. Across both experiments we found that compared to having no indication of when the probes would be presented, including visual cues to allow for anticipation of probes, led to reports of less mind-wandering and poorer performance on trials in a brief window immediately preceding the probe onset. Furthermore, in Experiment 2, we found that providing anticipatory cues for upcoming thought probes did not substantively influence people’s thoughts about aspects of the task. Taken together, these results are not easily reconcilable with the notions that the presence of visible probe markers motivated a refocusing of attention for better performance, or increased thoughts about task-related factors. Instead, our findings suggest that anticipation of an upcoming metacognitive evaluation diverts attentional resources away from both mind-wandering as well as performance.

The present results are consistent with recent work by Safati et al. (2024), demonstrating that attentional monitoring and control can be costly, leading to poorer performance, even when mind-wandering is reduced relative to normal rates. Evaluating one’s attention involves metacognitive processes, which may demand attentional resources (see Schooler, 2002). Our findings imply that the mere expectation of a metacognitive judgment appears to recruit cognitive resources, reducing the availability of these resources for both ongoing task performance and mind-wandering. This anticipation-related recruitment of cognitive resources may be driven by monitoring processes that operate outside of explicit awareness (see Schooler, 2002; Wegner, 1994). As a result, while participants may not consciously engage in active self-monitoring prior to probe onset, their attentional system appears to pre-emptively allocate resources in preparation for the expected introspective demand, leading to observable reductions in both mind-wandering and task performance.

Findings from the memory literature provide additional support for these interpretations. Research on prospective memory has demonstrated “the cost of remembering to remember,” whereby maintaining an intention to perform a future action imposes cognitive costs, even when that action is not immediately relevant (Cohen et al., 2008; McBride & Flaherty, 2020; Smith, 2003). Similarly, the “next-in-line effect” (Brenner, 1973; Caron et al., 2021; Forrin et al., 2019) suggests that when individuals anticipate an upcoming cognitive demand, their attention shifts away from processing ongoing stimuli, leading to impaired memory for events occurring immediately before the anticipated demand. These findings align with our results in that they also suggest that the anticipation of a cognitive event similarly redirects cognitive resources away from both task performance and mind-wandering, even if participants are not explicitly aware of this shift.

What brain networks might be involved in the anticipatory effects found in the present studies? Brain networks that underly attentional behaviors include the Salience Network (SN), which is thought to act as a switchboard between the Central Executive Network (CEN), and the Default Mode Network (DMN) (Menon, 2011; Seeley et al., 2007; Sridharan et al., 2008). While the CEN is typically associated with focus on the task at hand, the DMN is often associated with mind-wandering (Christoff et al., 2016). It has previously been observed that the presentation of salient stimuli activates the SN, suppressing the DMN, and activating the CEN (Sridharan et al., 2008). Given the dynamics of the SN, and the established relationship between mind-wandering and task performance, one might expect that the increasing salience of the anticipatory probe markers would also produce increased task performance. However, key nodes in the CEN, like the dorsolateral prefrontal cortex (DLPFC,) have been found to also play a key role in making metacognitive judgments (Vaccaro & Fleming, 2018). It is possible that as the brain anticipates having to perform expected metacognitive judgments, shared mental resources are split between the task and metacognitive evaluation without individuals having immediate conscious thoughts about the metacognitive judgment they will soon be performing.

Earlier cognitive models of control describe a Supervisory Attentional System (SAS) (Norman & Shallice, 1986), which can also provide an explanatory framework for our findings. Conceptually akin to the CEN, the SAS was thought to recruit regions in the prefrontal cortex for higher-order executive control, particularly in situations requiring novel judgment or the inhibition of automatic responses. From this conceptual framework, when participants anticipate an upcoming thought probe, engagement of the SAS may regulate attention to prepare for the metacognitive task. This perspective helps explain why we observed decreased mind-wandering but not improved task performance, as the SAS’s involvement in metacognitive preparation may come at the cost of engagement in the primary task.

We should also consider an alternative – though much less likely – interpretation of our findings. Thus far, our discussion has been based on the assumption that increasing attention to the primary metronome response task ought to improve performance, which should manifest as reduced response variability. This is a reasonable assumption based on prior use and interpretation of the task (Anderson et al., 2021; Laflamme et al., 2018; Safati et al., 2024; Seli, Cheyne, et al., 2013a, 2013b; Wilson et al., 2022). However, it is likely that low response variability in the task could result from a combination of controlled and automatic motor processes (Cheyne et al., 2012). Accordingly, there is the possibility that refocusing attention to the metronome task might disrupt the balance of these processes, resulting in poorer performance even though more attention is applied to the task. According to this view, both the subjective measure of attention (reduced mind-wandering) and the behavioral performance measure (increased response variability) would point to the anticipation of a probe reorienting attention to the primary task, perhaps based on increased task motivation driven by the anticipation of a probe. Future studies are required to better characterize the role of focal attention on performance in the metronome response task to evaluate this alternative explanation.

Several limitations warrant consideration. An important limitation concerns the possibility of temporal anticipation effects even when probes were not visible. Research on foreperiod effects has demonstrated that temporal preparation for upcoming events is influenced by the distribution and variability of intervals between warning signals and target stimuli (Karlin, 1959; Los et al., 2017; Niemi & Näätänen, 1981), raising the possibility that similar temporal learning processes may have occurred in our Not Visible conditions. More broadly, research on fixed and variable interval reinforcement schedules has shown that organisms develop temporal expectations when events occur at intervals, showing systematic changes in behavior as time elapses since the previous event (Catania & Reynolds, 1968; Gibbon, 1977; Killeen & Fetterman, 1988). In our experiments, participants in the Not Visible conditions knew probes would occur periodically, even though they could not predict the exact timing. It is therefore possible that, as time since the previous probe increased, participants may have begun to anticipate probe presentation, potentially producing effects similar to those in the Visible condition (i.e., reduced mind-wandering coupled with decreased performance). However, testing this hypothesis presents methodological challenges. The relationship between time since the previous probe and performance could reflect: (1) natural temporal increases in mind-wandering that occur even without anticipatory process (see Thomson et al., 2014; Zanesco et al., 2024); (2) the dissipating reorienting effect of the previous probe (Safati et al., 2024; Schubert et al., 2020); (3) emerging anticipation of the next probe based on learned temporal patterns; or (4) some combination of the above. Disentangling these competing processes would require careful statistical control and experimental designs specifically aimed at isolating anticipatory effects from the natural temporal dynamics of attention.

Beyond temporal learning considerations, another limitation is that our studies exclusively employed the metronome response task, which has some unique characteristics compared to other paradigms commonly used in mind-wandering research, such as reading comprehension, vigilance, and executive control tasks (Jackson & Balota, 2012; Rummel & Boywitt, 2014; J. Smallwood et al., 2009). One key difference is that other tasks often have critical trials (e.g., no-go trials, go trials, or target trials) that punctuate moments in the task, whereas the MRT more continuously measures rhythmic consistency across trials. Such differences may influence how anticipatory processes affect performance. As such, the generalizability of our findings to tasks with different cognitive demands remains an empirical question.

On a positive practical note, the present findings show that randomization of the presentation of experience sampling probes, a common practice in research on mind-wandering (e.g., Ju & Lien, 2018; Robison et al., 2019; Safati et al., 2024; Seli, Carriere, et al., 2013a, 2013b; Welhaf et al., 2023), appears to minimize anticipatory effects on task performance. While we cannot rule out that some temporal anticipation may develop even with unpredictable timing, performance decrements were substantially more pronounced in the trials leading up to explicitly signaled probes compared to those that were not. This suggests that reducing probe predictability, even if it cannot eliminate anticipatory effects entirely, may meaningfully attenuate their impact on task performance. This is consistent with prior findings indicating that the presence of experience sampling probes does not meaningfully influence task performance (Wiemers & Redick, 2019). Thus, reducing the predictability of thought probes appears to be a prudent practice. Studies that allow participants to anticipate the onset of probes should consider the potential consequences this can have on task performance and mind-wandering reports.

## Data Availability

Deidentified participant data along with the analysis script has been made available via the Open Science Framework at: https://osf.io/phjkq/?view_only=e521db50368b4295a939c66ce1db2162.

## Code availability

The code for the computer tasks used in both experiments has been made available via the Open Science Framework at: https://osf.io/phjkq/?view_only=e521db50368b4295a939c66ce1db2162.

## Declarations

### Open practices statements

The data, analysis code, and materials for both experiments are available at https://osf.io/phjkq/?view_only=e521db50368b4295a939c66ce1db2162. The experiments were not pre-registered.
